# Green self-accountability and temporal distance impact on sustainable consumption behavior: the mediating role of anticipated emotions

**DOI:** 10.3389/fpsyg.2025.1503642

**Published:** 2025-05-15

**Authors:** Bowei Zhong, Xingwang Feng, Xiaogang Li, Wei Fan

**Affiliations:** ^1^Institute of Psychology, Chinese Academy of Sciences, Beijing, China; ^2^Department of Psychology, University of Chinese Academy of Sciences, Beijing, China; ^3^Department of Psychology, Hunan Normal University, Changsha, China; ^4^Cognition and Human Behavior Key Laboratory of Hunan Province, Changsha, China; ^5^Institute of Interdisciplinary Studies, Hunan Normal University, Changsha, China

**Keywords:** green self-accountability, temporal distance, sustainable purchasing behavior, anticipated guilt, anticipated pride

## Abstract

**Purpose:**

With the rise in global consumption depleting natural resources and harming the environment, promoting sustainable consumption is essential. Our study explores how green self-accountability affects sustainable purchasing over different time frames, examining the impact on short (one week) and long-term (one year) choices toward green products and the underlying mechanisms.

**Methods:**

We conducted two experiments where participants made green purchasing decisions, with the first activating green self-accountability and the second also measuring anticipated emotional responses.

**Results:**

Findings show that green self-accountability boosts sustainable purchasing, particularly for immediate choices, driven by anticipated pride in buying green products.

**Conclusion:**

Green self-accountability significantly enhances sustainable purchasing, aided by anticipated pride. This suggests that marketing strategies emphasizing environmental urgency and consumers’ eco-unfriendly behaviors can foster sustainable consumer habits.

## 1 Introduction

The advent of the new century, with its marked increase in goods and services consumption, has drawn widespread attention to the resultant global depletion of natural resources and severe environmental damage (Ajibade and Boateng, 2021; Awewomom et al., 2024). Current global material consumption has reached 92.1 billion tons annually, doubling over the past four decades (Krausmann et al., 2017). Simultaneously, global carbon emissions have increased by half since the beginning of the 21st century, with the energy sector accounting for three-quarters of these emissions in 2020 [International Energy Agency (IEA), 2021]. In this critical context, a study by the Ellen MacArthur Foundation (2019) highlights that adopting circular economy principles emphasizing product longevity and recyclability could reduce global carbon emissions by 39% and virgin resource consumption by 28% by 2050. Advocacy for sustainable consumption, therefore, undeniably holds important contemporary significance (Moisander, 2007). Sustainable consumption involves consumers choosing environmentally friendly goods and services during purchase and usage – those that inflict minimal damage on the environment, are easily recyclable, have a longer lifespan, and do not pollute the environment, thus realizing sustainable utilization of resources and maintaining environmental harmony (Van der Werff et al., 2021).

In the 21st century, people’s consumption habits and choices have a profound impact on environmental conservation. Modern consumers no longer only focus on the quality, price, and popularity of products but also on their environmental attributes, supporting environmental protection through their consumption behavior (Moisander, 2007). This change in consumption concept requires time for consumers to gradually understand the impact of their consumption behavior on the environment and willingly adopt environmentally friendly practices in everyday life.

Green self-accountability, a significant element of individual identity recognition, further influences their purchasing decisions, thereby imbuing sustainable consumption with more social significance (Mishra et al., 2021). Green self-accountability refers to an individual’s aspiration to meet their environmental self-standards (Xia et al., 2022). Many have realized that environmental protection goals can be genuinely achieved only through collective effort. Previous research primarily focused on the relationship between green self-accountability and consumers’ sustainable choices (Nair and Little, 2016), showing a prominent positive impact of green self-accountability on purchasing green products. This demonstrates that consumer choice goes beyond product quality, price, and popularity to a greater emphasis on environmental attributes (Pedersen, 2000).

Despite the increasing focus on environmental sustainability, consumers still face considerable challenges in choosing green products that have higher environmental performance but lower popularity (Ali et al., 2023). This is largely due to such products often failing to cater to their needs for individual satisfaction and social recognition (Connolly and Prothero, 2008). For instance, some green products may be priced higher or their designs may not meet consumer esthetic standards, leading to hesitation in purchasing green products.

Consumers experience a cognitive dissonance or internal conflict when forced to choose between satisfying personal needs and purchasing environmentally friendly products. It is essential, therefore, to delve deeper into the psychology and behaviors of consumers faced with such internal conflict, exploring the role of temporal distance and anticipated emotional reactions in fostering green self-accountability and driving purchasing behavior. Temporal distance refers to individuals’ perception of how far an event is from the present. It influences people’s preferences, judgments, and behavioral responses by affecting their construal level of events or objects, playing a significant role in consumers’ purchase intentions (Trope and Liberman, 2000). When consumers are in a distant temporal perspective, corresponding to a high construal level, their personal environmental self-standards become more salient. They experience a stronger sense of connection between their actions and both others’ welfare and environmental protection. Consequently, their desire to fulfill environmental self-standards intensifies, thereby promoting green purchasing behavior (Wu et al., 2015; Kaiser and Wilson, 2004). Green self-accountability forms the intrinsic motivation behind sustainable consumption behaviors, and anticipated emotional reactions may influence expectations about purchasing green products, thereby indirectly affecting their purchase decisions.

Overall, this study aims to investigate the impact of green self-accountability and temporal distance on purchasing environmentally-friendly products with less popularity, and reveal the mediating effect of anticipated guilt and pride on the interaction between green self-accountability and temporal distance. Compared to existing research, the unique contribution of this study lies in its pioneering integration of temporal distance into the research framework examining green self-accountability and purchasing behavior, revealing its crucial role in moderating consumer decision-making. For instance, while Tran and Paparoidamis' (2021) study primarily focused on the direct relationship between self-accountability and consumers’ sustainable choices without considering the influence of temporal distance, and Trope and Liberman's (2000) research investigated the impact of temporal distance on behavioral decision-making without connecting it to green consumption behavior, the current study bridges this research gap by integrating green self-accountability with temporal distance. Furthermore, it elucidates the mediating mechanism of anticipated emotional responses. These findings not only deepen our understanding of green consumption behavior but also provide theoretical foundations for businesses to design marketing strategies across different temporal frameworks.

Research suggests that self-accountability plays a critical role in influencing consumers’ purchase behaviors for green products (Tran and Paparoidamis, 2021). Consumers with an established environment-conscious identity tend to assume responsibility for their purchasing choices, motivating them toward the acquisition of green products (Alagarsamy et al., 2021). A heightened sense of personal responsibility has been found to positively impact consumers’ ecological behaviors, particularly evident during green purchases (Gatersleben et al., 2002).

Previous research has explored how an individual’s sense of environmental responsibility impacts their green buying behaviors. Findings indicate a strong positive correlation between an individual’s sense of environmental responsibility and their environmentally-friendly purchasing activity. This suggests that responsibility acts as a predictor for green consumer behavior (Jung and Cho, 2023). Research by Naparin and Astuti (2025) indicated that consumers’ awareness and concern regarding environmental issues could potentially amplify their likelihood of making environmentally friendly purchases. Van der Werff et al. (2014) have similarly suggested that consumers displaying an established sense of environmental responsibility are more likely to engage in green buying behavior.

On the flip side, consumers who believe environmental responsibility resides with others or organizations are more inclined toward non-green consumption. Khare’s (2015) research underscores the relationship between green self-accountability and environmentally friendly purchasing behavior. It suggests green self-accountability may act as a key influencer of consumers’ environmentally friendly purchasing behavior, a notion supported by data from Indian consumers. Elsantil’s (2021) study similarly identified a positive relationship between green self-accountability and environmentally friendly purchasing behavior, especially in the Arabian Gulf region. The study’s contribution mainly lies in revealing the relationship between consumer’s green purchasing behavior, green self-accountability, social influence, and eco-labeling.

In conclusion, the proposition that green self-accountability enhances consumers’ likelihood of purchasing green products holds, as substantiated by ample supporting studies.

*Hypothesis 1*: Green self-accountability can enhance consumers’ green purchases. Consumers with a heightened sense of environmental responsibility are likely prone towards making green decisions as they believe they hold a duty to contribute towards environmental preservation.

Under recent temporal distance, green self-accountability can effectively enhance consumers’ green purchasing behavior. This could be due to consumers perceiving their green purchasing behavior having a significant positive effect on the environment, thus further enhancing their enthusiasm toward purchasing green products (Walker et al., 2014; Steg et al., 2015). Consumers may view their behavior of purchasing green products as a manifestation of them upholding environmental responsibility (Nguyen et al., 2024), and believe their actions can directly contribute to the improvement of environmental quality (Kaplan et al., 2024). Under near temporal distance, consumers’ green self-accountability may be further enhanced by the observable positive effects of environmentally friendly actions (Kollmuss and Agyeman, 2002), thereby promoting environmentally focused purchasing decisions (Verplanken and Holland, 2002). Additionally, other studies have discovered that consumers’ green self-accountability may impact their consumption choices (Luchs et al., 2010; De Groot and Steg, 2009), and that they are more willing to accept higher prices when purchasing green products (Pickett‐Baker and Ozaki, 2008).

*Hypothesis 2:* Under recent temporal distance, green self-accountability more effectively enhances consumers’ green purchasing behavior. This is because consumers realize that their green purchasing behavior can immediately have a positive impact on the environment, thereby enhancing their enthusiasm for green purchases.

According to research by Mishra et al. (2024), future-oriented emotions of self-pride and guilt play a crucial role in affecting environmentally friendly behaviors, particularly in the realm of green purchases. These emotions may elevate consumers’ expectation of their actions either having a positive (pride) or negative (guilt) repercussion (Dwyer et al., 2015). Additionally, research by Haj-Salem et al. (2022) suggested these expected emotions of pride and guilt play a part in shaping consumers’ receptivity to green products – a correlation that’s amplified when consumers possess a high level of environmental responsibility and contemplate environmental behaviors within a proximal timeframe.

Similarly, Nascimento and Loureiro (2024) highlighted that consumer’s anticipated guilt could enhance their intent to evade non-environmental actions, subsequently driving them toward green purchases. Ultimately, research by Chae et al. (2024) revealed that consumers’ anticipated pride and moral responsibility could boost their willingness to purchase organic products. Overall, anticipated feelings of pride and guilt could serve as integral internal elements in the interplay of environmental responsibility and temporal distance in impacting green purchasing behavior.

*Hypothesis 3:* Anticipated feelings of pride and guilt play a mediating role in the impact of green self-accountability and temporal distance on green purchasing behavior. Specifically, consumers with a strong sense of green self-accountability and proximity in temporal distance may experience an increase in anticipated pride, potentially promoting more green purchases. On the other hand, consumers possessing a high level of green self-accountability, and considering the long-term negative impacts of failing to purchase green products, may experience increased anticipated guilt, possibly escalating their green purchases.

In conclusion, anticipated feelings of pride and guilt might serve as a significant mechanism within the dynamic of green self-accountability and temporal distance on green buying activity. These emotions may simultaneous occur alongside considerations of environmental responsibilities and future implications during purchasing decisions, resulting in more eco-friendly acquisitions.

## 2 Experiment 1

### 2.1 Methods

#### 2.1.1 Participants and experimental design

Our study applied a 2 (priming: green self-accountability priming vs. control) × 2 (temporal distance: short vs. long) between-participants experimental design. The dependent variable involved participants’ purchase intention toward green products (shower gel, laundry detergent). The required sample size was computed using G*Power software (power = 0.8, effect size *f* = 0.25, *α* = 0.05), amounting to 179 participants. The sample consisted of 180 university students, 42 of whom were males. All participants possessed normal or corrected-to-normal vision. Assignment of participants to the various experimental conditions was randomized. Participants’ age ranged between 18 to 25 years.

#### 2.1.2 Experimental materials

##### 2.1.2.1 Green self-accountability priming materials

Participants in the green self-accountability group were asked to complete a series of scrambled-sentence tasks (Chen and He, 2017). First, participants were asked to complete a task involving the rearrangement of 10 sentences. They were required to choose four out of five disordered words to form a fluent and reasonable sentence and write it down. For instance, the unordered words “ecology,” “last,” “important,” “is,” “very” could be reassembled to form the sentence “Ecology is very important.” After the sentence formulation task, five target detection words, such as “waste,” “attention,” “quality,” “improve,” “cherish,” were presented to the priming group. Subsequently, participants were instructed to add the first character or word that came to mind before or after each of the five target detection words, forming a meaningful word or phrase.

To balance out the potential disruptive influence of additional factors, the control group also implemented the scrambled-sentence task. Participants in this group were required to complete ten sentence construction tasks unrelated to environmental sustainability, which involved directly forming words or phrases using the five target detection words.

##### 2.1.2.2 Manipulation of green products type

Product Material: Regular products were defined as having low eco-friendly and high popularity attributes, while green products were defined as having high eco-friendliness and low popularity attributes, which represent the key information of both product types.

The environmental attribute of a product was defined as its environmental protection index. The environmental protection index refers to the evaluation of the environmental friendliness of similar products (like biodegradability, no damage to water quality, etc.) by the International Environmental Assessment Committee. In the formal experiment, subjects were informed about the product’s environmental protection index (keeping other product information consistent), where an index of 5, the research’s low eco-friendly level, represents an average level among similar products, and an index of 10, the research’s high eco-friendly level, represents the highest level among similar products (Wang et al., 2017, 2018; Wang and Wu, 2018).

The product popularity attribute was defined by the product’s sales in the last month on internet e-commerce platforms (participants are informed that this is the actual sales volume).

In this experiment, the key information for the regular shower gel product was an environmental index of 5 and a monthly sales volume of 1,127 (representing its popularity). The key information for the green shower gel product was an environmental index of 10 and a monthly sales volume of 8. The regular laundry detergent product had key attributes of an environmental index of 5 and a monthly sales volume of 1,105, while the green version had key attributes of an environmental index of 10 and a monthly sales volume of 6.

##### 2.1.2.3 Manipulation of temporal distance

A fundamental mechanism underlying the temporal effects in human decision-making is that people’s predictions about the future depend on their mental representations of future environments (Liberman et al., 2002). In accordance with past research, temporal distance was set as one week and one year scenarios for purchase (Wang et al., 2017; Wang et al., 2018; Wang and Wu, 2018).

Participants were asked to imagine that due to life circumstances, they need to purchase a shower gel (or laundry detergent) product after a week (or a year).

#### 2.1.3 Experimental procedure

The data was gathered via the “Questionnaire Star” platform, with participants conducting the study on computers within the lab environment uniformly.

Participants first completed the green self-accountability priming.

Next, they were involved in a decision-making scenario involving the purchase of shower gel (or laundry detergent). They were first asked to visualize a situation in which, due to life circumstances, they would have to buy a green product (shower gel or laundry detergent) a week or a year later. Afterwards, the key information about the regular and green products was presented to the participants. Finally, they were asked to make a relative purchase intention evaluation between regular and green products.

A 1 to 9 scale representing regular products to green products was used. If the participants were more inclined to purchase regular products, the purchase intention rating leaned toward 1. If they preferred to purchase green products, the rating would lean toward 9. This allowed the extraction of the participants’ relative purchase intentions toward green products.

### 2.2 Results and discussion

#### 2.2.1 Results of manipulation check for experimental products

To validate the effectiveness of the product popularity manipulation, we selected 44 participants who did not take part in the main experiment to evaluate the attributes of the two products, shower gel and laundry detergent, using a 7-point semantic differential scale. The results, as shown in Table 1, verified that the manipulation of product popularity was effective. Significant differences were observed between low-popularity and high-popularity products, regardless of whether they were shower gels or laundry detergents.

**Table 1:** Comparison of the mean product familiarity in the experiment with the average familiarity.

Product type	Popularity	*M ± SD*	*t*(*N* = 43)	*p*
Shower	High	5.23 ± 1.12	8.91	<0.001
	Low	2.80 ± 1.42		
Laundry detergent	High	5.48 ± 0.95	9.03	<0.001
	Low	2.86 ± 1.61		

#### 2.2.2 Results of operation test on environmental responsibility

The count of environmentally related words added before and after the probe word by the two groups of participants was statistically analyzed. Descriptive outcomes are presented in Table 2. Environmental-related words included air, water, water resources, environment, resources, etc.

**Table 2:** Number of environmental words filled by two groups of subjects (*M ± SD*).

Experimental group	*M ± SD*	*t*(*N* = 26)	*p*
Environmental self-accountability	2.23 ± 1.65	8.14	<0.001
Control	0.61 ± 0.86		

An independent samples t-test suggested that the participants in the environmental self-accountability priming group enlisted significantly more environmentally related words than the control group, with *t* (179) = 8.14, *p* < 0.001. These findings support the effectiveness of the environmental self-accountability priming operation.

#### 2.2.3 Purchasing intention

In order to reduce error, a 2 (Priming type: Environmental Self-Accountability priming, Control Group) × 2 (Time distance: Near-term purchase, Long-term purchase) × 2 (Product type: Shower gel, Detergent) repeated measures analysis of variance was conducted on the purchasing intentions for shower gel and detergent. The results indicated a significant interaction between priming type and time distance, *F*(1, 176) = 11.44, *p* = 0.001, *η_p_^2^* = 0.06.

Through simple effect analysis, it was found that for the purchasing intention of shower gel, the effect of priming type was significant under the near-term time distance, *F*(1, 177) = 17.97, *p* < 0.001. Compared with the control group, participants primed with environmental self-accountability showed a stronger willingness to purchase green products. In the long-term time distance, the effect of the priming type was not significant, *F* < 1.

For the purchasing intention of detergent, the effect of priming type was significant under the near-term time distance, *F*(1, 177) = 26.46, *p* < 0.001. Similar to the shower gel, participants from the environmental self-accountability priming group were more inclined to buy green products than those in the control group. Under the long-term time distance, the effect of the priming type was also not significant, *F* < 1.

The interaction between product type and priming type was not significant, *F*(1, 176) = 1.79, *p* = 0.183; the interaction between product type and time distance was not significant, *F*(1, 176) = 0.76, *p* = 0.384; the three-way interaction between product type, time distance, and priming type was also not significant, *F* < 1.

The main effect of the priming type was significant, *F*(1, 176) = 12.38, *p* = 0.001, *η_p_^2^* = 0.07. Post-hoc tests showed that the purchasing intention of participants in the environmental self-accountability priming group (6.21 ± 0.259) was greater than that of the participants in the control group (4.94 ± 0.251).

**Figure 1:**
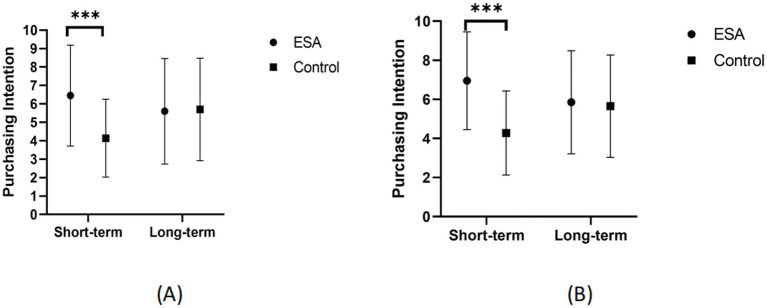
Green products purchase intention (***p < 0.001; ESA, environmental self-accountability). **(A)** Shower gel. **(B)** Laundry detergent.

The main effect of time distance was not significant, *F*(1, 176) = 0.46, *p* = 0.500. The main effect of product type was significant at the marginal level, *F*(1, 176) = 2.95, *p* = 0.088, *η_p_^2^* = 0.02. The results are shown in Table 3 and Figure 1.

**Table 3:** Green product purchase intention (*M ± SD*).

Product type	Experimental group	Temporal distance	*M ± SD*
Shower gel	Environmental self-accountability	Short-term	6.45 ± 2.74
		Long-term	5.60 ± 2.86
	Control	Short-term	4.14 ± 2.11
		Long-term	5.70 ± 2.78
Laundry detergent	Environmental Self-Accountability	Short-term	6.95 ± 2.50
		Long-term	5.85 ± 2.64
	Control	Short-term	4.28 ± 2.15
		Long-term	5.65 ± 2.62

The results of Experiment One validated Hypotheses One and Two, affirming that activating environmental self-accountability can effectively enhance individuals’ purchasing intention of green products. Particularly in the short-term timeframe, participants in the environmental self-accountability priming group showed a stronger willingness to purchase green products compared to the control group. Also, the study results indicate that in the short-term timeline, participants in the environmental self-accountability priming group have a higher tendency to buy green products than those in the control group. The study found a significant increase in the willingness to buy green products among participants whose environmental self-duty was stimulated in a near-term purchasing decision scenario.

In Experiment One, we explored the impact of environmental self-accountability and time distance on green consumption. However, we did not specifically discuss the mechanisms through which environmental self-accountability and time distance affect green consumption. Therefore, in Experiment Two, we will investigate Hypothesis Three, examining the mediating role of anticipated emotions in the influence of environmental self-accountability and time distance on green consumption.

## 3 Experiment 2

### 3.1 Methods

#### 3.1.1 Participants and experimental design

This experiment adopted a 2 (Priming Type: Environmental Self-Accountability Priming, Control Group) × 2 (Time Distance: short-term, Long-term) between-subjects experimental design. The dependent variable was the participants’ willingness to purchase green products (shower gel, laundry detergent, refrigerator, air conditioner). The required number of participants was calculated using G*Power software (power = 0.8, effect size *f* = 0.25, *α* = 0.05) yielding a necessary sample size of 179 participants. However, we recruited 180 participants, including university students and members of the general public, of which 86 were male. All the participants had normal or corrected-to-normal vision. The participants were randomly assigned to either the priming group or the control group. The age of participants ranged from 17 to 62 years, with an average age of 30.53 (*SD* = 7.951) years and a median age of 30 years.

#### 3.1.2 Experimental materials

Green self-accountability priming materials: same as in experiment 1. Manipulation of temporal distance: same as in experiment 1. Manipulation of green products type.

In this experiment, the green products included the two types of products used in Experiment 1, with the addition of two more common green products used in daily life, namely refrigerators and air conditioners. The presentation of information for the green and ordinary products in Experiment 2 was the same as in Experiment 1.

Therefore, the key information in this experiment were as follows:

- The environmental index of the ordinary shower gel product was 5, with a monthly sales volume of 1,127 units. The environmental index of the green shower gel was 10, with a monthly sales volume of 8 units.
- The environmental index of the ordinary laundry detergent product was 5, with a monthly sales figure of 1,105 units. The environmental index of the green laundry detergent was 10, with a monthly sales figure of 6 units.
- The environmental index for the ordinary refrigerator product was 5, with a monthly sales volume of 1,043 units. The environmental index of the green refrigerator was 10, with a monthly sales volume of 10 units.
- The environmental index of the ordinary air conditioner product was 5, with a monthly sales volume of 1,178 units. The environmental index of the green air conditioner was 10, with a monthly sales figure of 18 units.

#### 3.1.3 Measure of emotions

Measurement of expected emotions: In this experiment, the expected emotions being measured were anticipated guilt and pride. We used Anticipated Guilt Rating questionnaire (*α* = 0.92, in this research *α* = 0.93) and Anticipated Pride Rating questionnaire (*α* = 0.93, in this research *α* = 0.89) to measure the participants’ expected feelings of guilt and pride after making decisions (Peloza et al., 2013; Rowe et al., 2017).

#### 3.1.4 Experimental procedure

The procedure for Experiment 2 was largely similar to Experiment 1, with the main difference being that Experiment 2 needed to measure the participants’ expected emotions. For this purpose, the participants were asked to fill out an expected emotion scale after they had made their purchasing inclination assessment.

### 3.2 Results and discussion

#### 3.2.1 Experimental product manipulation check results

Since two new products were added, we selected 22 participants who did not participate in the formal experiment to rate the popularity of the two products, refrigerators and air conditioners, on a 7-point semantic scale. The results of the check were as follows Table 4.

**Table 4:** Statistical analysis of product popularity.

Product type	Popularity	*M ± SD*	*t*(*N* = 21)	*p*
Refrigerator	High	5.64 ± 0.85	8.97	<0.001
	Low	2.41 ± 1.05		
Air conditioner	High	5.77 ± 0.75	7.95	<0.001
	Low	2.68 ± 1.323		

Participants’ choices for less popular products, such as refrigerators and air conditioners, were significantly lower compared to highly popular products. The results indicate that the popularity manipulation of the two products chosen in the experiment was effective.

#### 3.2.2 Environmental self-accountability manipulation check results

The number of environment-related words in the words added before and after the test words were statistically analyzed for both groups of participants. The control group used significantly fewer environment-related words than the environmental group, indicating the successful manipulation of green self-accountability activation between the two groups.

The descriptive results are shown in Table 5.

**Table 5:** Number of environmental words filled out by the two groups of participants.

Experimental group	*M ± SD*	*t*(*N* = 179)	*p*
Environmental self-accountability	2.81 ± 1.68	9.15	<0.001
Control	0.82 ± 1.17		

#### 3.2.3 Purchasing intention

**Figure 2:**
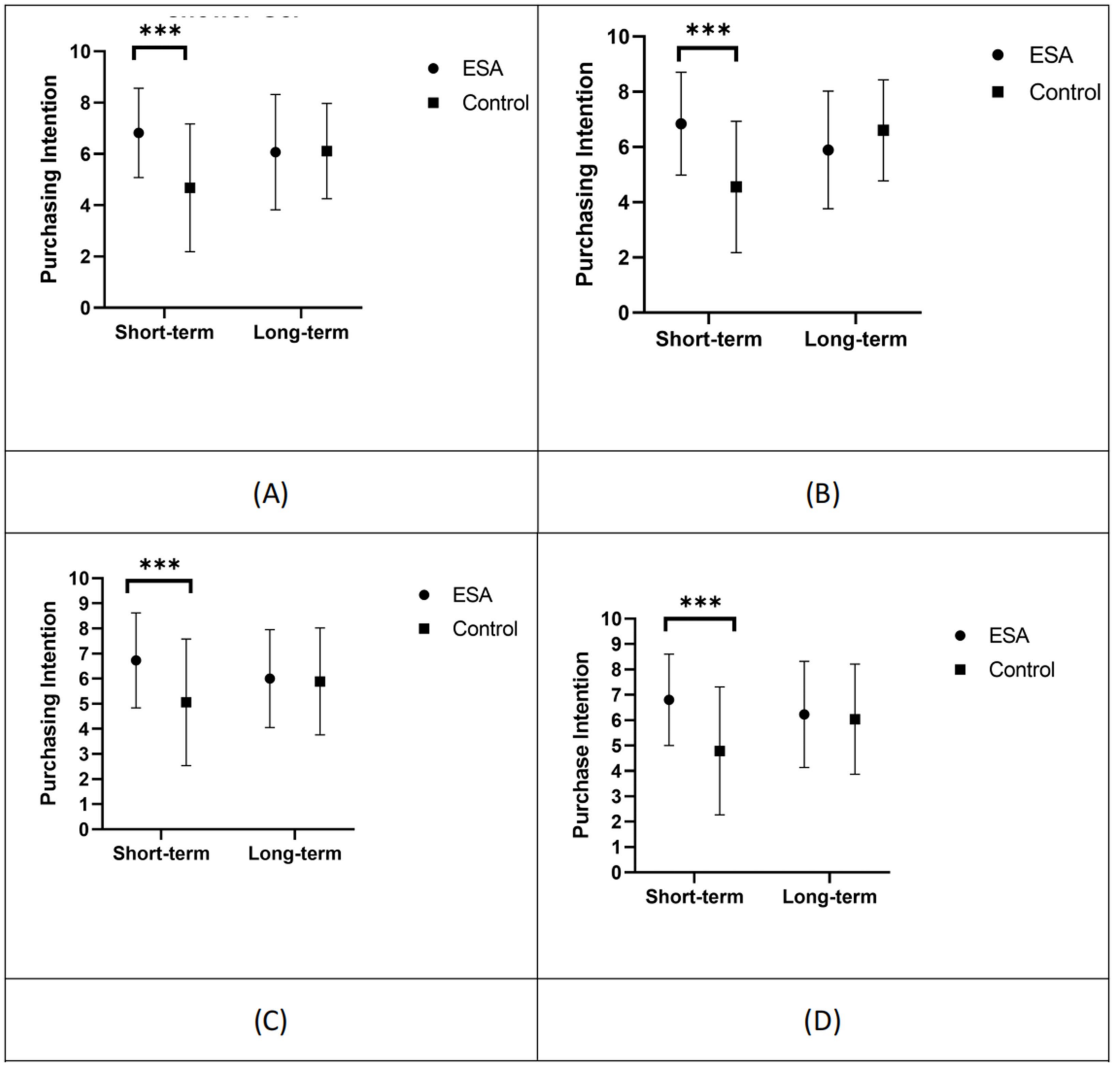
High eco-friendly products purchase intention (***p < 0.001; ESA, environmental self-accountability). **(A)** Shower gel. **(B)** Laundry detergent. **(C)** Refrigerator. **(D)** Air conditioner.

To minimize errors, a 2 (Priming Type: Environmental Self-Accountability Priming, Control Group) × 2 (Temporal Distance: Short-term, Long-term) × 4 (Product Type: Shower Gel, Laundry Detergent, Refrigerator, Air Conditioner) repeated measures ANOVA was conducted on purchase intention toward products. The results indicated a significant interaction between self-accountability and temporal distance, *F*(1, 176) = 15.31, *p* < 0.001, *η_p_^2^* = 0.08. The interaction between product type and self-accountability was not significant, *F* < 1. The interaction between product type and temporal distance was not significant, *F*(3, 176) = 1.24, *p* = 0.296. However, the three-way interaction among product type, self-accountability, and temporal distance was significant, *F*(3, 176) = 2.92, *p* = 0.034, *η_p_^2^* = 0.016. A significant main effect of self-accountability was found, *F*(1, 176) = 12.18, *p* = 0.001, *η_p_^2^* = 0.065, with post-hoc analysis revealing that the purchase intention of participants in the self-accountability priming condition (6.42 ± 0.195) was higher than that of the control group participants (5.47 ± 0.191). The main effect of temporal distance was not significant, *F*(1, 176) = 1.37, *p* = 0.244. The main effect of product type was also not significant, *F* < 1. The results are illustrated in Figure 2 and Table 6. Following the research objectives, a simple effect analysis of the three-way interaction among product type, self-accountability, and temporal distance is as follows:

**Table 6:** Descriptive statistics for green product purchase intention.

Product type	Experimental group	Temporal distance	*M ± SD*
Shower gel	Environmental self-accountability	Short-term	6.82 ± 1.74
		Long-term	6.07 ± 2.25
	Control	Short-term	4.68 ± 2.49
		Long-term	6.11 ± 1.86
Laundry detergent	Environmental self-accountability	Short-term	6.84 ± 1.86
		Long-term	5.89 ± 2.13
	Control	Short-term	4.55 ± 2.38
		Long-term	6.60 ± 1.83
Refrigerator	Environmental self-accountability	Short-term	6.73 ± 1.89
		Long-term	6.00 ± 1.95
	Control	Short-term	5.06 ± 2.52
		Long-term	5.89 ± 2.13
Air conditioner	Environmental self-accountability	Short-term	6.80 ± 1.80
		Long-term	6.23 ± 2.09
	Control	Short-term	4.79 ± 2.52
		Long-term	6.04 ± 2.17

For the purchase intention of shower gels: under short temporal distance, the priming type effect was significant, *F*(1, 177) = 23.48, *p* < 0.001, indicating that compared to the control group, participants primed with self-accountability had a stronger intention to purchase green products (shower gels). Under long temporal distance, the priming type effect was not significant, *F* < 1.

For the purchase intention of laundry detergents: under short temporal distance, the priming type effect was significant, *F*(1, 177) = 28.07, *p* < 0.001, indicating that compared to the control group, participants primed with self-accountability had a stronger intention to purchase green products (laundry detergents). Under long temporal distance, the priming type effect was not significant, *F*(1, 177) = 2.68, *p* = 0.104.

For the purchase intention of refrigerators: under short temporal distance, the priming type effect was significant, *F*(1, 177) = 13.77, *p* < 0.001, indicating that compared to the control group, participants primed with self-accountability had a stronger intention to purchase green products (refrigerators). Under long temporal distance, the priming type effect was not significant, *F* < 1.

For the purchase intention of air conditioners: under short temporal distance, the priming type effect was significant, *F*(1, 177) = 19.73, *p* < 0.001, indicating that compared to the control group, participants primed with self-accountability had a stronger intention to purchase green products (air conditioners). Under long temporal distance, the priming type effect was not significant, *F* < 1.

#### 3.2.4 The mediating role of anticipated emotion

##### 3.2.4.1 The mediating role of anticipated guilt

In this study, Mplus7.0 software and bootstrap method were utilized to further identify the mediating role of anticipated guilt between environmentally responsible behavior, temporal distance, and green purchase intention. The results showed that the model fit well, with parameters: Bootstrap = 1,000, CFI = 1.00, TLI = 1.00, RMSEA = 0.00.

**Figure 3:**
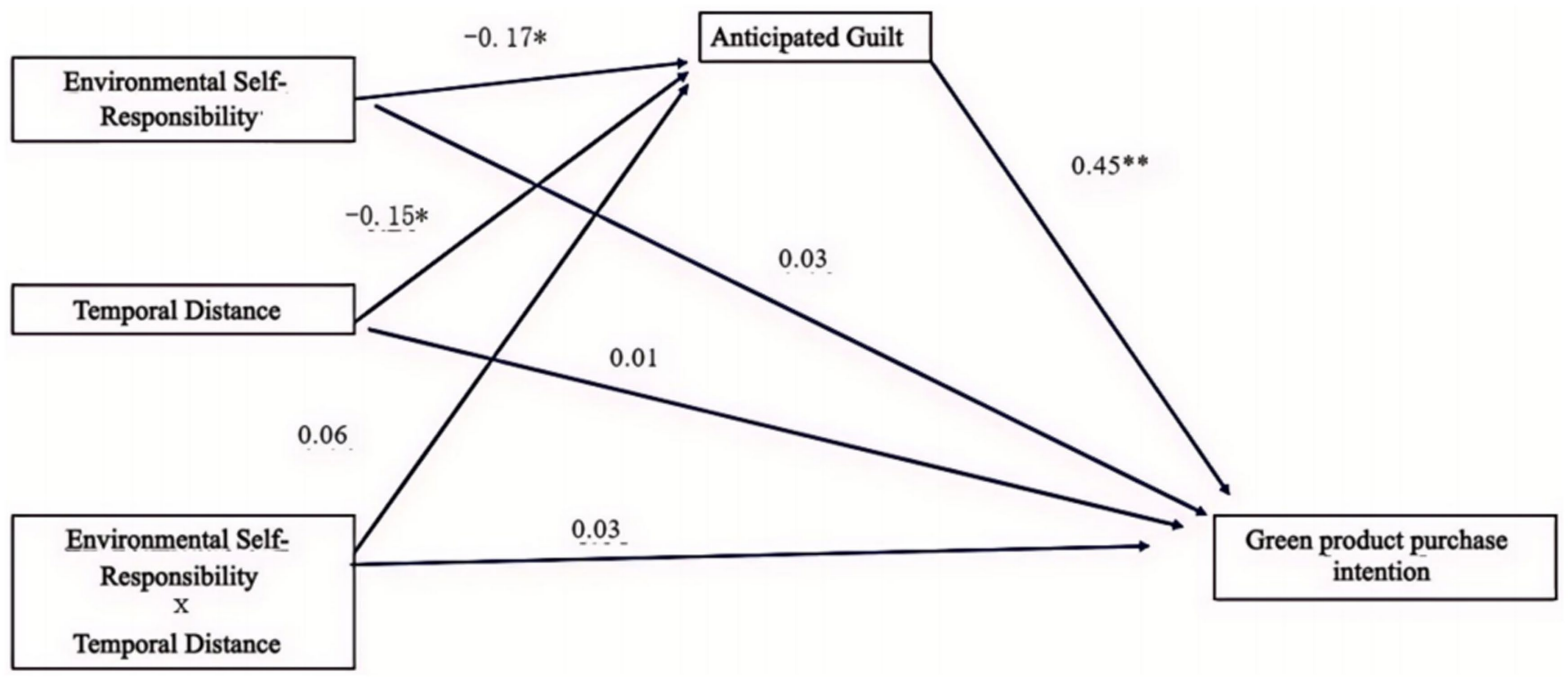
Path analysis between anticipated guilt, environmental self-duty, time distance, and green buying intention (*p < 0.05, ***p < 0.001).

The path diagram is illustrated in Figure 3. The effect analysis is detailed in Table 7.

**Table 7:** Path analysis among anticipated guilt, environmental responsibility, temporal distance, and green purchase intention.

Effect	*β*	Ratio
ESA → AG → GPI	0.077^*^	53.5%
TD → AG → GPI	0.067^*^	46.5%

*p < 0.05, **p < 0.01, ***p < 0.001. ESA, Environmental self-accountability; AG, Anticipated guilt; TD, Temporal distance; GPI, Green purchase intention.

As shown in Figure 3 and Table 7, we discovered that anticipated guilt played a complete mediating role between environmental self-accountability and green purchase intention (*β* = 0.077, *p* < 0.05). Furthermore, anticipated guilt also played a full mediation role between temporal distance and green purchase intention (*β* = 0.067, *p* < 0.05). However, anticipated guilt did not mediate between the moderating role of environmental self-accountability and temporal distance, and green purchase intention (*p* > 0.05). These results provide strong evidence for our understanding of the relationship between environmental self-accountability, temporal distance, and green purchase intention.

##### 3.2.4.2 The mediating role of anticipated pride

While further exploring the mediating role of anticipated pride between environmental self-accountability, temporal distance, and green purchase intention, we employed Mplus7.0 software and the Bootstrap method. The model’s goodness of fit was extraordinary, with specific parameters as follows: Bootstrap = 1,000, CFI = 1.00, TLI = 1.00, RMSEA = 0.00.

**Figure 4:**
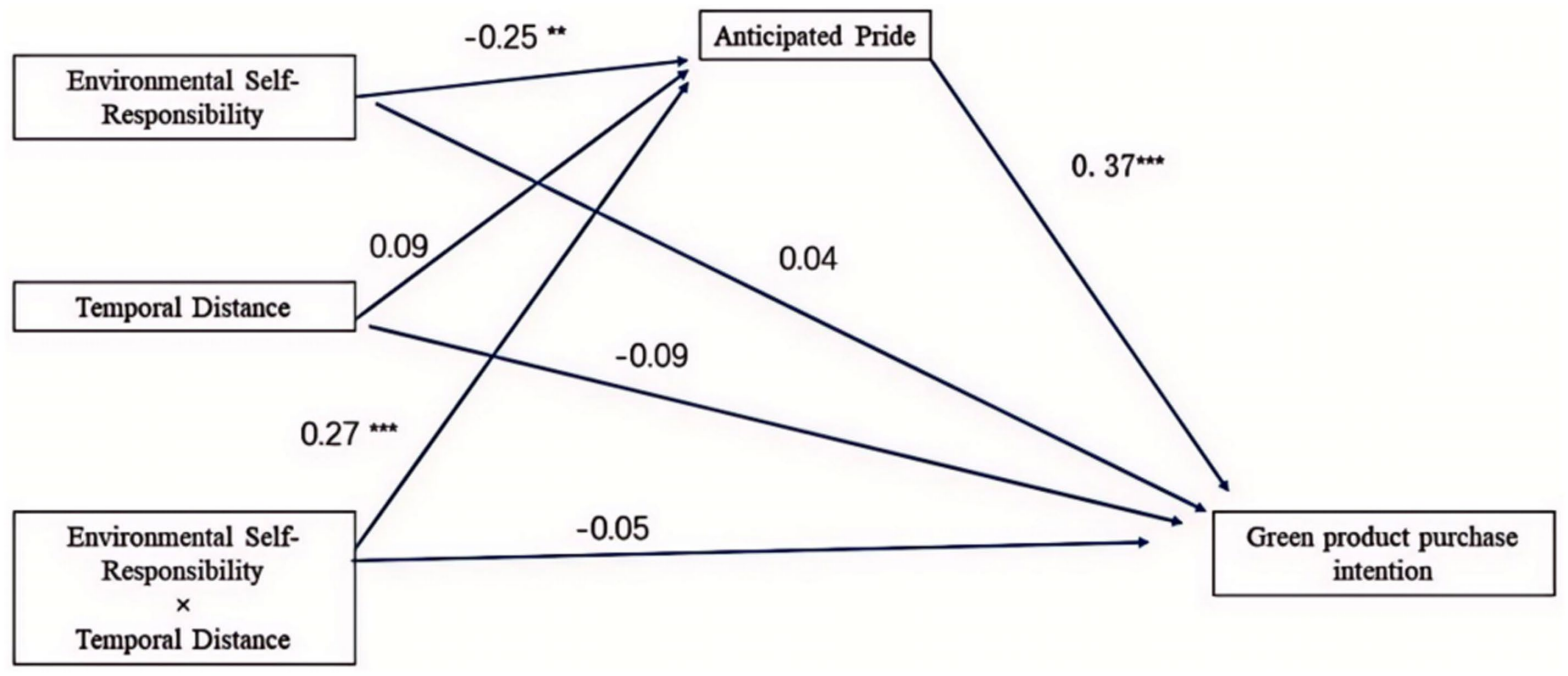
Path analysis between anticipated pride, environmental self-duty, time distance, and green buying intention (**p < 0.01, ***p < 0.001).

From the results data in Figure 4 and Table 8, it is clear that anticipated pride fully mediates the relationship between environmental self-accountability and green purchase intention (*β* = 0.092, *p* < 0.001). However, anticipated pride did not show a mediating effect between temporal distance and green purchase intention (*p* > 0.05). Nevertheless, we discovered that anticipated pride does indeed mediate comprehensively between the moderating effect of environmental self-accountability and temporal distance, and green purchase intention (*β* = 0.102, *p* < 0.001).

**Table 8:** Effect analysis between anticipated pride, environmental self-accountability, temporal distance, and green purchase intention.

Effect	*β*	Ratio
ESA → AP → GPI	0.092^***^	47.4%
ESA × TD → AP → GPI	0.102^***^	52.6%

***p < 0.001; ESP, Environmental self-accountability; AP, Anticipated pride; TD, Temporal distance; GPI, Green purchase intention.

Experiment two again confirmed the results of experiment one, namely that environmental self-accountability can promote the purchase of green products, and the activation of environmental self-accountability can effectively promote the participants’ recent purchase of green products. At the same time, we found that both anticipated guilt and anticipated pride play a mediating role in the impact of environmental self-accountability on green consumption. However, only anticipated pride has a mediating role in the interaction between environmental self-accountability and temporal distance. This outcome suggests that the role of environmental self-accountability in boosting an individual’s green purchase intention under short temporal distance is achieved through anticipated pride.

## 4 General discussion

This research delved deeply into the role of environmental self-accountability and temporal distance on the intention to purchase green products. Our subjects comprised of university students, and the study focused on readily available products like shower gel and laundry detergent.

The results underscore that the activation of environmental self-accountability significantly enhances buyer’s intentions, thus corroborating our hypothesis. This observation reinforces the established findings of previous research that individuals mindful of environmental conservation prefer green products (Peloza et al., 2013; Wu, 2014). Furthermore, we observed that the activation of environmental self-accountability drastically influences short purchasing decisions, aligning with the theories of Liberman and Trope (1998) and Wakslak et al. (2008) that people prioritize personal gain when decision-making revolves around immediate purchases. A deeper analysis of the interaction effects reveals that green self-accountability and temporal distance jointly influence green purchase intention. Specifically, the interaction effect is significant for low-involvement products but not for high-involvement products. This finding aligns with construal level theory, which posits that temporal distance shapes individuals’ perception and evaluation of products (Trope and Liberman, 2010). For low-involvement products, the immediacy of purchase decisions amplifies the effect of green self-accountability, whereas for high-involvement products, the extended decision-making process attenuates this effect. This nuanced understanding underscores the importance of simultaneously considering psychological and contextual factors in green consumption research.

In the pursuit of broadening our understanding, we examined the impact of environmental self-accountability on other types of green products. Household appliances like refrigerators and air conditioners were our focus in the second part of our experiment. The results again confirmed that the activation of environmental self-accountability significantly promotes the intention to purchase green products in the near future; thereby strengthening our theoretical hypothesis.

Importantly, our research attempted to demystify the underlying mechanism of environmental-self responsibility. We proposed anticipated emotion as a possible influencing factor, which aligns to the studies by Aaker et al. (2010). Predicted emotion – the preset emotional response to future outcomes, significantly influence purchase behavior. Our data showed that activating environmental self-accountability markedly elevates the anticipated pride of consumers, consistent with earlier studies on anticipated emotions’ impact on purchase decisions (Zeelenberg et al., 2008; Liu et al., 2013).

In summary, by demonstrating the interaction between green self-accountability, temporal distance, and product type, this study extends the understanding of green self-accountability. The findings indicate that activating green self-accountability significantly enhances green purchase intention across different product types, including low-involvement products (e.g., body wash and laundry detergent) and high-involvement products (e.g., refrigerators and air conditioners). However, the strength of this effect varies by product type. For low-involvement products, the impact of green self-accountability on purchase intention is more pronounced in the short term, likely due to their lower cost and higher purchase frequency, which aligns with the construal level theory (Liberman and Trope, 1998; Wakslak et al., 2008). In contrast, for high-involvement products, this effect is more gradual, possibly due to the greater financial commitment and extended decision-making process (Thøgersen, 2005). These findings suggest that marketers should tailor their strategies based on product type, emphasizing immediate benefits for low-involvement products and long-term environmental impacts for high-involvement products.

Furthermore, this study highlights the mediating role of anticipated emotions, providing a psychological mechanism for the observed effects. Practically, our findings suggest that marketers can enhance green purchase intentions by evoking anticipated pride and guilt. For instance, advertising campaigns could emphasize the positive self-image associated with green choices or the negative consequences of environmentally harmful behaviors (Schultz et al., 2007). Policymakers could also leverage these insights to design interventions that promote sustainable consumption.

However, this study has several limitations. First, the main effect of temporal distance on green product choices was not significant. The nonsignificant impact of temporal distance on green consumption behavior may be attributed to the nature of the products examined. For low-involvement products, the immediacy of purchase decisions may obscure the effect of temporal distance, whereas for high-involvement products, the long-term benefits of green products may mitigate the influence of temporal distance (Griskevicius et al., 2012). Additionally, individual differences in time orientation may play a role, as some individuals prioritize long-term environmental benefits regardless of temporal distance (Zimbardo and Boyd, 1999). Future research should explore these factors to better understand the conditions under which temporal distance influences green consumption.

Second, regarding the emotional mediation, our study confirms that anticipated pride and guilt mediate the relationship between green self-accountability and green purchase intention. However, the underlying mechanisms of these emotions require further investigation. For example, anticipated pride may enhance self-efficacy, motivating individuals to align their behaviors with environmental values (Bandura, 1997). In contrast, anticipated guilt may act as a deterrent against environmentally harmful behaviors by triggering avoidance of negative self-evaluation (Tangney et al., 2007). Future research should examine how these emotions interact with other psychological factors, such as social norms and personal values, to provide a more comprehensive understanding of the mediation process.

Another limitation of this study is the reliance on a university student sample, which may constrain the generalizability of the findings. The green consumption behaviors of university students may be influenced by their educational background and values. Therefore, future studies should include more diverse samples encompassing different age groups, income levels, and cultural backgrounds to enhance external validity (Henrich et al., 2010). Additionally, the ecological validity of the experimental design could be improved by incorporating real purchase data or field experiments. For example, future research could track actual green product purchases to validate the findings derived from hypothetical scenarios.

Finally, this study selected product types with considerable differences in price range and purchase frequency (e.g., shampoo, laundry detergent, refrigerators, air conditioners) in Experiments 1 and 2, which may have influenced the results. Low-involvement products (e.g., shampoo and laundry detergent) are relatively inexpensive and frequently purchased, leading consumers to focus more on immediate benefits in decision-making. In contrast, high-involvement products (e.g., refrigerators and air conditioners) are more expensive and less frequently purchased, prompting consumers to prioritize long-term benefits (Thøgersen, 2005). These product type differences may have contributed to variations in the effects of green self-accountability and temporal distance on purchase intention. Future research should further control for product type effects, for example, by selecting products with similar price levels and purchase frequencies for comparison, to more accurately reveal the underlying mechanisms of green self-accountability.

Despite several limitations like the demographic of our sample, variability in products etc., our study provides fresh insights into the impact of environmental self-accountability on green consumer behavior and provides empirical evidence for future similar studies in the field. The findings attest the importance of environmental self-accountability in enhancing green product purchases, offering robust theoretical evidence for enterprises promoting green products, particularly environmental marketing strategies. To further understand the mechanistic impact of environmental self-accountability, future research would require an in-depth investigation of its relationship with anticipated emotions, along with potential influential factors.

## 5 Conclusion

The current study explored the influence of environmental self-accountability and temporal distance on the intention to purchase green products utilizing an experimental approach, and validated the mediating role of anticipated emotions within this context. The findings denote that sparking environmental self-accountability significantly amplifies an individual’s intention to purchase green products, notably in the context of a short temporal distance. In addition to this, we discovered that anticipated pride entirely mediates between environmental self-accountability and the intention to purchase green products, further revealing the operational mechanisms of environmental self-accountability.

This investigation offers valuable insights into the impact of environmental self-accountability on green purchase intention, providing theoretical support and practical guidance for environmental behavior and promotion.

## Data Availability

The datasets presented in this study can be found in online repositories. The names of the repository/repositories and accession number(s) can be found in the article/supplementary material.
